# Supplementary material for: Multi‐dataset identification of innovative feature genes and molecular mechanisms in keratoconus
Source: J Cell Mol Med. 2024 Sep 19;28(18):e70079. doi: 10.1111/jcmm.70079 (PMC11412914; doi:10.1111/jcmm.70079)
Supplement: Supplementary file 2 — Material S2. [file JCMM-28-e70079-s002.zip › 7.GSEA analysis/6.SNORA74B/KEGG_TYROSINE_METABOLISM.html]

Details for gene set KEGG\_TYROSINE\_METABOLISM[GSEA]

|  || Dataset | SNORA74B.group.cls#h\_versus\_l |
| Phenotype | group.cls#h\_versus\_l |
| Upregulated in class | l |
| GeneSet | KEGG\_TYROSINE\_METABOLISM |
| Enrichment Score (ES) | -0.4743575 |
| Normalized Enrichment Score (NES) | -1.5405152 |
| Nominal p-value | 0.007984032 |
| FDR q-value | 0.3366988 |
| FWER p-Value | 0.712 |
Table: GSEA Results Summary

  

Fig 1: Enrichment plot: KEGG\_TYROSINE\_METABOLISM      
 Profile of the Running ES Score & Positions of GeneSet Members on the Rank Ordered List

  

| SYMBOL | TITLE | RANK IN GENE LIST | RANK METRIC SCORE | RUNNING ES | CORE ENRICHMENT || 1 | AOC2 | na | 747 | 0.534 | 0.0377 | No |
| 2 | TAT | na | 1441 | 0.422 | 0.0645 | No |
| 3 | MAOB | na | 1824 | 0.387 | 0.0965 | No |
| 4 | ADH1B | na | 4400 | 0.246 | 0.0496 | No |
| 5 | ADH6 | na | 4824 | 0.231 | 0.0630 | No |
| 6 | ADH4 | na | 5073 | 0.223 | 0.0806 | No |
| 7 | IL4I1 | na | 5832 | 0.198 | 0.0807 | No |
| 8 | PNMT | na | 6780 | 0.169 | 0.0721 | No |
| 9 | TPO | na | 7938 | 0.138 | 0.0540 | No |
| 10 | TRMT11 | na | 8766 | 0.119 | 0.0434 | No |
| 11 | TH | na | 11370 | 0.069 | -0.0240 | No |
| 12 | METTL2B | na | 12061 | 0.059 | -0.0373 | No |
| 13 | GOT2 | na | 12459 | 0.054 | -0.0428 | No |
| 14 | GSTZ1 | na | 12554 | 0.052 | -0.0397 | No |
| 15 | ALDH3B1 | na | 12635 | 0.051 | -0.0363 | No |
| 16 | DDC | na | 15228 | 0.022 | -0.1086 | No |
| 17 | ADH7 | na | 15231 | 0.022 | -0.1061 | No |
| 18 | FAH | na | 15329 | 0.021 | -0.1066 | No |
| 19 | MAOA | na | 16871 | 0.006 | -0.1504 | No |
| 20 | COMT | na | 18119 | -0.007 | -0.1855 | No |
| 21 | LCMT1 | na | 18403 | -0.011 | -0.1925 | No |
| 22 | LCMT2 | na | 18881 | -0.016 | -0.2045 | No |
| 23 | MIF | na | 18896 | -0.016 | -0.2031 | No |
| 24 | GOT1 | na | 19922 | -0.028 | -0.2296 | No |
| 25 | ALDH3A1 | na | 24095 | -0.082 | -0.3407 | No |
| 26 | ADH1A | na | 25803 | -0.113 | -0.3774 | No |
| 27 | METTL6 | na | 26663 | -0.132 | -0.3876 | No |
| 28 | ADH5 | na | 26984 | -0.139 | -0.3814 | No |
| 29 | HEMK1 | na | 27506 | -0.151 | -0.3796 | No |
| 30 | ALDH3B2 | na | 27617 | -0.154 | -0.3656 | No |
| 31 | AOX1 | na | 31382 | -0.287 | -0.4422 | Yes |
| 32 | DCT | na | 32497 | -0.354 | -0.4351 | Yes |
| 33 | AOC3 | na | 32580 | -0.359 | -0.3975 | Yes |
| 34 | HPD | na | 32828 | -0.378 | -0.3627 | Yes |
| 35 | ALDH1A3 | na | 32981 | -0.391 | -0.3236 | Yes |
| 36 | DBH | na | 33323 | -0.427 | -0.2861 | Yes |
| 37 | HGD | na | 33487 | -0.448 | -0.2411 | Yes |
| 38 | ADH1C | na | 34198 | -0.581 | -0.1971 | Yes |
| 39 | TYRP1 | na | 34624 | -0.831 | -0.1171 | Yes |
| 40 | TYR | na | 34712 | -1.082 | 0.0005 | Yes |
Table: GSEA details [plain text format]

  

Fig 2: KEGG\_TYROSINE\_METABOLISM      
 Blue-Pink O' Gram in the Space of the Analyzed GeneSet

  

Fig 3: KEGG\_TYROSINE\_METABOLISM: Random ES distribution      
 Gene set null distribution of ES for **KEGG\_TYROSINE\_METABOLISM**
